# Phytochemicals and Antioxidant Activities of Red Oak, Red Coral and Butterhead

**DOI:** 10.21315/tlsr2023.34.1.1

**Published:** 2023-03-31

**Authors:** Nadechanok Jiangseubchatveera, Charinrat Saechan, Arpa Petchsomrit, Tawikan Treeyaprasert, Nattawut Leelakanok, Chadaporn Prompanya

**Affiliations:** 1 Division of Pharmacognosy and Pharmaceutical Chemistry, Faculty of Pharmaceutical Sciences, Burapha University, Bangsaen, Muang, Chonburi 20131, Thailand; 2 Faculty of Medical Technology, Prince of Songkla University, Hat Yai, Songkhla 90110, Thailand; 3 Department of Pharmaceutical Technology, Faculty of Pharmaceutical Sciences, Burapha University, Bangsaen, Muang, Chonburi 20131, Thailand; 4 Research Unit of Pharmaceutical Innovations of Natural Products (PhInNat), Burapha University, Chonburi 20131, Thailand; 5 Department of Mathematics and Statistics, Faculty of Science and Technology, Thammasat University, Pathumthani 12121, Thailand; 6 Department of Clinical Pharmacy, Faculty of Pharmaceutical Sciences, Burapha University, Chonburi, Bangsaen, Muang, Chonburi 20131, Thailand

**Keywords:** Phytochemical Screening, Phenolic Content, Flavonoid Content, Antioxidant Activity, Lettuce

## Abstract

*Lactuca sativa* L. is an economically important vegetable that contains numerous phytochemicals. This study aimed to determine the phytochemicals in three lettuce cultivars (red oak, red coral, and butterhead) and assess their total phenolics, total flavonoids and antioxidant activities. The dried leaves of each lettuce cultivar were macerated with hexane, ethyl acetate (EtOAc), and 95% ethanol (EtOH). Total phenolics, total flavonoids, and antioxidant activities from the three solvent extracts were measured. The phytochemical screening showed that the leaves from the three lettuce cultivars contained flavonoids, hydrolyzable tannins, coumarins, steroids, and phenolic compounds. While the EtOAc fraction of the red coral lettuce showed the highest total phenolic content (9.747 ± 0.021 mg gallic acid equivalent/g) and the hexane fraction of the butterhead lettuce contained the highest flavonoids (7.065 ± 0.005 mg quercetin equivalent/g). In the DPPH (2,2-diphenyl-1-picryl-hydrazyl-hydrate) assay, the EtOAc fraction of the red coral lettuce had the highest antioxidant capacity with an IC_50_ of 0.277 ± 0.006 mg/mL, whereas, in the ABTS (2,2′-azino-bis(3-ethylbenzothiazoline-6-sulfonic acid)) assay, the 95% EtOH of the red coral lettuce had the highest antioxidant capacity with an IC_50_ of 0.300 ± 0.002 mg/mL. All three lettuce cultivars contained high levels of phenolic content and flavonoids, which are the source of antioxidant activities. These lettuce cultivars, especially the red coral lettuce, are a potential source of natural antioxidants. Further research on the application of natural antioxidants is required to investigate the therapeutic or the neutraceutical implication of the lettuce cultivars.

**Highlights**
- The leaves from the three lettuce cultivars contained flavonoids, hydrolyzable tannins, coumarins, steroids and phenolic compounds.
- The EtOAc fraction of the red coral lettuce and the hexane fraction of the butterhead lettuce showed the highest total phenolic content and the highest flavonoid content, respectively.
- The red coral lettuce had the highest antioxidant capacity using the DPPH and ABTS assays.

## INTRODUCTION

Vegetables are excellent source of essential nutrients and fibers, which provide health benefits. *Lactuca sativa* L., a plant commonly known as lettuce, is in the *Asteraceae* family. Lettuce can be crudely categorised using head formation, leaf shape, texture and stem type (Kim *et al*. 2016). Most commonly consumed lettuce are crisphead, butterhead, romaine and leaf lettuce. Crisphead lettuce has crisp round green leaves that form a compact head. Butterhead lettuce has sweet soft leaves that form one round non-compact head. While romaine or cos lettuce forms an upright elongated non-compact head. Leaf or cutting lettuce, e.g., red oak and red coral, has crisp leaves that are arranged on the stalk loosely. Lettuce leaf is usually green, although red lettuce exists. This leads to a vast variety of lettuce appearances.

Because of the diversity in the texture and flavour of lettuce, it is considered the most popular salad plant (Llorach *et al*. 2008). Lettuce can be consumed fresh, with preserved nutritional value because heat exposure is bypassed (Kim *et al*. 2016). Lettuce, including crisphead, butterhead, romaine, green oak and red oak, is particularly rich in antioxidants, e.g., phenolic acids, flavonoids, anthocyanins, vitamins C and vitamin E (Kim *et al*. 2016; Lanza Volpe *et al*. 2021). These compounds are heat-labile (Ross *et al*. 2011; Ioannou *et al*. 2020; Mori *et al*. 2007; Vikram *et al*. 2005). Antioxidants react with oxidative free radicals, e.g., hydroxyl radical, superoxide anion radical and peroxynitrite radicals, which are unstable and highly reactive molecules that react with macromolecules, e.g., nucleic acids, proteins, carbohydrates and lipids which are found in the nucleus and cells (Maddu 2019; Lobo *et al*. 2010). Studies have shown that antioxidants prevent cell injury from free radicals and might be beneficial in reducing risks of various diseases including cardiovascular diseases, cancer, Alzheimer’s disease and renal diseases (Aune *et al*. 2018; Jayedi *et al*. 2018). The extract of lettuce is available as a health supplement product that claims health benefits based on antioxidant activity. Other health benefits of lettuce include anti-inflammatory (Mulabagal *et al*. 2010; Younus *et al*. 2019), analgesic (Younus *et al*. 2019) and anticancer potential (Zhou *et al*. 2019; Qin *et al*. 2018).

In spite of lettuce’s potential for the nutraceutical industry due to its antioxidant activity, several challenges remain. Due to the variation of lettuce, one of the challenges is the variation in antioxidants yield. Types and parts of lettuce confer different antioxidant activities (Gan & Azrina 2016; Ozgen & Sekerci 2011). Investigation of the antioxidative profile and yield from lettuce is useful for the development of lettuce extract-based products. However, despite the widespread study of the antioxidant activity in natural plants, the study on the antioxidant activity of lettuce is scarce. Investigating lettuce from various sources enables the comparison of the contents and antioxidant activity of lettuce to literature. Therefore, this research aimed to qualitatively identify the phytochemicals and to quantitatively determine the total phenolics, total flavonoids and the antioxidant activities from dried leaves of three common lettuce cultivars (red oak, red coral and butterhead) in Thailand.

## MATERIALS AND METHODS

### Materials

All organic solvents were analytical grade. For instance, hexane, ethyl acetate (EtOAc), acetone, ethanol (EtOH), methanol (MeOH), dichloromethane (CH_2_Cl_2_), sodium hydroxide (NaOH), ammonium hydroxide (NH_4_OH), potassium hydroxide (KOH), potassium iodide (KI), sodium chloride (NaCl), glacial acetic acid, nitric acid and hydrochloric acid (HCl) were obtained from RCI Labscan (Bangkok, Thailand). While sulfuric acid (H_2_SO_4_), and ammonia solution were purchased from Carlo Erba (France). Then there’s the Folin-Ciocalteu’s reagent, sodium carbonate (Na_2_CO_3_), aluminum chloride (AlCl_3_), ferric chloride (FeCl_3_), hydrogen peroxide (H_2_O_2_), gelatin and acetic anhydride, which were purchased from Merck (Darmstadt, Germany).

Diphenylpicrylhydrazyl (DPPH), and bismuth subnitrate were purchased from Sigma-Aldrich (St. Louis, USA). 2,2′-Azino-bis(3-ethylbenzothiazoline-6-sulfonic acid) (ABTS), potassium persulfate, 6-hydroxy-2,5,7,8-tetramethyl-chroman-2-carboxyligac acid (Trolox) and 3,5-dinitrobenzoic acid were purchased from Aldrich (Milwaukee, USA). L-ascorbic acid and lead acetate were obtained from Ajax Finechem (Seven Hills, Australia). Quercetin and gallic acid were purchased from TCI (Tokyo, Japan). Lime water, bromine water, α-naphthol, tannic acid, sodium iodide, bismuth carbonate, picric acid, mercuric chloride, cadmium iodide and vanillin were purchased from LOBA Chemie (Mumbai, India).

### Sample Preparation and Extraction

Three cultivars of lettuce (*Lactuca sativa* L.): red lettuce (red oak and red coral) and green lettuce (butterhead) were collected from Wang Nam Khiew Organic Farms Network, Nakhon Ratchasima, Thailand. The leaves of red oak, red coral and butterhead were washed and dried in a hot air oven at 50°C for 24 h. Then, the ground dried leaves (50.0 g) were extracted in 0.6 L of each solvent at room temperature for 24 h in the following order: hexane, EtOAc and 95% EtOH. In other words, the total extraction time for the sample was 72 h. Each extract was then filtered and evaporated under reduced pressure using a rotary evaporator (Buchi, Rotavapor^®^ R-210, Germany) to obtain crude hexane extracts, crude EtOAc extracts, and crude EtOH extracts. All samples were stored at 4°C until further analysis.

### Phytochemical Testing

The different extracts of lettuce were screened for the constituents of secondary metabolites using the following standard procedures.

#### Flavonoids (Sittisombut 2010)

- Shinoda’s test: A small piece of magnesium was added to 1 mL of 95% ethanolic extract followed by adding approximately two to three drops of concentrated hydrochloric acid. The development of a pink, reddish or brown colour after a few minutes indicated the presence of flavonoids.
- Molisch’s test: A milliliter of 95% ethanolic extract was mixed with three to four drops of 5% α-naphthol followed by two to three drops of concentrated H_2_SO_4_. A layer of violet coloration was formed at the interface which indicated the presence of glycosides.

#### Tannins (Sittisombut 2010)

- True tannins: The mixture of 5 mL of 95% ethanolic extract and 5 mL of water was added to three to four drops of 10% NaCl solution and then the volume was adjusted to 10 mL. Five milliliters of the mixture were mixed with 10 drops of 1% gelatin solution. The other 5 mL of the mixture was used as a control. The appearance of the stable sediments indicated the presence of true tannins.
- Condensed tannins: Three milliliters of 95% ethanolic extract were dried. Three drops of vanillin TS were then added and observed for the appearance of intense red colour which indicated the presence of condensed tannins.
- Hydrolyzable tannins: Two milliliters of 95% ethanolic extract were mixed with 2 mL of water. The lime water (1–2 mL) was then added and observed for grayish-yellow sediments which indicate the presence of hydrolyzable tannins.

#### Coumarins (Sittisombut 2010)

The screening of coumarins was divided into two groups:

- Volatile coumarins detection: The dried sample (1 g) was moistened and transferred into a test tube. Then, the tube was sealed with the filtered paper treated with 10% NaOH solution and heated for 5 min. Next, the filter paper (Whatman Filter Paper, Sigma-Aldrich, USA) was removed and observed. The presence of volatile coumarins was detected by the presence of a greenish-blue fluorescence under the exposure to 365 nm ultraviolet (UV) light (Spectroline^®^ CM UV-viewing cabinet, Supelco, USA).
- Non-volatile coumarins detection: The 95% ethanolic extract was dropped onto a filter paper soaked with 10% NaOH solution. The presence of non-volatile coumarins was detected by the appearance of a greenish-blue fluorescence under 365 nm UV light.

#### Saponins (Soonthornchareonnon 2011)

The dried sample (1 g) was extracted with 15 mL of boiling water. The aqueous extract was filtered and cooled. Two milliliters of the aqueous extract were then added with 2 mL of water. This 4 mL solution was prepared for two tubes and was used for the following tests:

- The first tube was shaken vigorously and observed for the formation of stable persistent froth. This indicated the presence of saponins.
- A milliliter of 5% HCl solution was added to the second tube and then shaken vigorously. The formation of a honeycomb-like froth that remained for 10 min indicated the presence of saponins.

#### Anthraquinones (Soonthornchareonnon 2011)

The dried sample (1 g) was mixed with 10% KOH (20 mL) and 3% H_2_O_2_ solution. The mixture was refluxed for 10 min, filtered, and adjusted the pH with acetic acid to pH 3–4. The pH-adjusted solution was partitioned with CH_2_Cl_2_ (10 mL). The CH_2_Cl_2_ extract was then added to the NH_4_OH solution. The appearance of a pinkish-red colour in a basic layer indicated the presence of anthraquinones.

#### Cardiac glycosides (Soonthornchareonnon 2011)

The mixture of 10 mL of 95% ethanolic extract and 10 mL of water was partitioned with 10 mL of CH_2_Cl_2_. The CH_2_Cl_2_ crude extract was used for the following tests:

- Kedde’s test: Three milliliters of the CH_2_Cl_2_ crude extract were dried and mixed with Kedde’s reagent. The appearance of a reddish-violet colour indicated the presence of unsaturated lactone.
- Liebermann-Burchard test: Three milliliters of the CH_2_Cl_2_ crude extract were dried and mixed with acetic anhydride (2 drops) and concentrated H_2_SO_4_ solution. The appearance of a greenish-blue or red colour indicated the presence of a steroidal nucleus.
- Keller-Killani test: Three milliliters of CH_2_Cl_2_ extract were added into 2 mL of 10% FeCl_3_ in glacial acetic acid, followed by the addition of 2–3 drops of the concentrated H_2_SO_4_ solution. At the interface, a greenish-blue or brown ring was then formed when deoxysugar of cardiac glycosides was in the sample.

Three positive test results indicated the presence of cardiac glycosides in the extract.

#### Phenolics (Sittisombut 2010)

A milliliter of 95% ethanolic extract was mixed with a few drops of 1% FeCl_3_ solution. The presence of phenols caused the development of blue, green, or blackish-brown colours.

#### Alkaloids (Soonthornchareonnon 2011)

A few drops of 95% ethanolic extract were mixed with 5% HCl and then treated separately with different reagents; Dragendorff’s, Marme’s, Mayer’s, Wagner’s, and Tannic acid, respectively. The positive results were demonstrated after a few minutes by the formation of orange, white, yellowish-white, reddish-brown and yellowish-white precipitates, respectively. Three positive test results indicated the presence of alkaloids in the extract.

### Determination of Total Phenolic Content

Total phenolic content was determined by the Folin-Ciocalteu assay (Deetae *et al*. 2012). Briefly, different concentrations of the fractions in MeOH were prepared. The sample solution (300 μL) was mixed with 1.5 mL of 10% Folin-Ciocalteu’s reagent and 1.2 mL of 7.5% w/v Na_2_CO_3_. The mixture was left in the dark for 30 min at room temperature. Next, the absorbance was measured utilising a UV/Visible Spectrophotometer (Hitachi, U-2900, Japan) at a wavelength of 765 nm against a blank without extract. Gallic acid (0.01–0.07 mg/mL) in methanol was used as standard. The experiment was performed in triplicate for each concentration. All data were expressed as milligram gallic acid equivalents per gram of the extract (mg GAE/g) by comparing with the standard curve of gallic acid.

### Determination of Total Flavonoid Content

We modified the method by Ramamoorthy and Bono (2007) to determine the total flavonoid content of the fractions. A milliliter of a sample (10 mg/mL in MeOH) was mixed with 1 mL of 2% AlCl_3_ in MeOH. After incubation for 10 min at room temperature, the absorbance was then measured by the UV/Visible Spectrophotometer at 415 nm against a blank sample (1 mL of sample solution with 1 mL of MeOH). A standard solution of quercetin in methanol was prepared with a concentration of 0.001–0.07 mg/mL. Triplicate determinations were performed. The total flavonoid content was calculated from a quercetin calibration curve, and the result was expressed as mg quercetin equivalent per gram of the extract (mg QE/g).

### Antioxidant Assays

The antioxidant activity of all extracted samples was analysed in triplicate by DPPH and ABTS assays.

#### DPPH radical scavenging activity

The free radical scavenging activity of the extracts was measured for the antioxidant activity using the DPPH method (Jiangseubchatveera *et al*. 2017). The DPPH is a very stable free radical because of the delocalisation of free electrons on the whole molecule so the formation of the dimer does not occur. The DPPH radical scavenging capacity assay is used to quantify the ability of antioxidants in quenching the DPPH radical. In the presence of antioxidants, the colour of DPPH radical changes from dark purple to yellow.

In this experiment, all extract solutions were prepared in ethanol at various concentrations (1.0–10.0 mg/mL). Twenty microliters of each concentration were added to 180 μL of freshly DPPH in ethanol (0.0066% w/v) in a 96-well microtiter plate. The mixtures were incubated in the dark at room temperature for 30 min. After that, the absorbance was measured using spectrophotometry at a wavelength of 520 nm (Multi-mode microplate reader, FLUOStar Omega 2020, Germany). Trolox and ascorbic acid were used as reference standards. The percentage of DPPH scavenging activity was calculated as; [(A_control_ – A_sample_)/ A_control_] ×100; where A_control_ is the absorbance of blank and A_sample_ is the absorbance of the sample or standard at 520 nm. DPPH radical scavenging activity is expressed as the concentration of a sample required to decrease DPPH absorbance by 50% (IC_50_).

#### ABTS radical scavenging activity

The free radical scavenging activity of three lettuce cultivars was determined using ABTS radical cation (ABTS^•+^) decolourisation assay (Turapra *et al*. 2016) with some modifications. The ABTS assay is a colorimetric assay based on the ABTS cation radical (ABTS^•+^) formation is reduced by antioxidants, resulting in the change of colour from pale greenish to colourless.

Briefly, 5 mL of 7 mM ABTS were mixed with 2.5 mL of 2.4 mM potassium persulfate. The solution was stored in the dark at room temperature for 16 h to allow the generation of ABTS^•+^. The freshly prepared ABTS^•+^ solution was diluted with deionised water and ABTS^•+^ concentration was determined by a spectrophotometer (Multi-mode microplate reader, FLUOStar Omega 2020, Germany). The proper absorbance range of prepared ABTS^•+^ standard solution was 0.700 ± 0.020. The samples were prepared in EtOH at various concentrations (0.1 mg/mL–50 mg/mL). Twenty microliters of sample were then added to 180 μL of the ABTS^•+^ solution in a 96-well microtiter plate and left at room temperature for 5 min. Finally, the absorbance at 734 nm was measured. Trolox and ascorbic acid in a concentration range of 0.5 nM to 2.5 mM were used as the standards.

The percentage of ABTS inhibition (ABTS inhibition %) was calculated as [(A_control_ – A_sample_)/ A_control_] × 100; where A_control_ is the absorbance of the control reaction (containing all reagents except the test sample) and A_sample_ was the absorbance of the test sample. The IC_50_ values denote the concentration of the sample which is required to scavenge 50% of free radical by ABTS^•+^.

### Statistical Analysis

The data were reported as the mean ± standard deviation. The differences among total phenolics, total flavonoids, DPPH radical scavenging assay and ABTS radical scavenging assay were analysed by one-way analysis of variance. All statistical analysis was performed using GraphPad Prism 5 (GraphPad Software, USA). A *p*-value of less than 0.05 was considered statistically significant.

## RESULTS AND DISCUSSION

### Phytochemical Testing

Table 1 shows the results of the phytochemical analysis of the three lettuce cultivars (red oak, red coral and butterhead). The common phytochemistry content from the lettuce include flavonoids, hydrolyzable tannins, phenolics, steroids and volatile and non-volatile coumarins. First, red coral, red oak and butterhead lettuce cultivars contained steroidal nucleus (Liebermann-Burchard test). This agrees with the study from Mughrbi *et al*. (2020) which also found steroids in romaine and iceberg lettuce cultivars. Steroids in lettuce are not likely to be cardiac glycosides because the Keller-Killani test for deoxy sugar in cardiac glycosides was negative. Second, we found aglycone flavonoids in the lettuce. Literature reported that flavonoids in lettuce are isohamnetin, quercetin, kaempferol, epicatechin, mycertin and anthocyanin (Mampholo *et al*. 2016). Next, Tri-4-hydroxyphenylaceticacid-glucoside is the only tannin reported in the publication (Viacava *et al*. 2018). Moreover, common lettuce phenolics are caffeic acid derivatives, predominantly chicoric, chlorogenic, caffeoyltartaric and caffeoylmalic acids. Also, flavonol glycosides including quercetin 3-*O*-malonylglucoside, quercetin 3-*O*-glucoside (Talubnak *et al*. 2017), and quercetin 3-*O*-glucuronide (Ferreres *et al*. 1997) are found in lettuce. In addition, red varieties contain cyanidin-3-*O*-β-glucopyranoside, cyanidin-3-*O*-(6″-malonyl-β-glucopyranoside) and methyl ester of cyanidin-3-*O*-(6″-malonyl-β-glucopyranoside) (Mulabagal *et al*. 2010). Last, the finding of coumarins, both volatile and non-volatile, in these three lettuce cultivars is supported by other studies that also identified coumarins in lettuce (Viacava *et al*. 2018). These compounds are well known for their antioxidant activities and contribute to the radical scavenging capabilities of lettuce (Kim *et al*. 2016; Mughrbi *et al*. 2020; Mampholo *et al*. 2016; Materska *et al*. 2019).

### Total Phenolic and Flavonoid Content

Phenolic compounds are important secondary metabolites with antioxidant activity. The hydroxyl groups are responsible for free radical scavenging. The total phenolic content (TPC) investigated using the Folin–Ciocalteu reagent is presented in Table 2. The statistical analysis of these results exhibited significant differences in TPC among three lettuce varieties (*p* < 0.05), except for the EtOH extracts of red coral and butterhead. Among the three solvents, the EtOAc extracts of all three cultivars of lettuce showed the highest TPC while hexane extracts contained the lowest TPC. The order was similar to the TPC content in *Aurea helianthus* flowers extract (EtOAc > EtOH > hexane) (Ri *et al*. 2019). In other words, in this study, EtOAc was the best solvent for the extraction of TPC. This is because polar solvents, e.g., ethyl acetate and methanol can extract phenolic chemicals better than nonpolar solvents (Vittaya *et al*. 2019). We also found that the amounts of total phenolics differ among the three lettuce cultivars. The EtOAc fraction of red coral showed the highest TPC (9.747 mg GAE/g). Studies supported that red lettuce exhibits higher antioxidant activity than green lettuce (Llorach *et al*. 2008; Liu *et al*. 2007) because red pigments in the lettuce are anthocyanins which are phenolics (Liu *et al*. 2007). In addition, a review by Kim *et al*. (2016) showed that red leaf and red oak lettuce had the greatest level of phenolics while crisphead green lettuce had low levels of phenolics.

The basic quantitative determination of the total flavonoid content (TFC) in the extracts of three lettuce determined by aluminum chloride in a colorimetric method is presented in Table 2. The TFC from three lettuce cultivars ranged from 1.093–7.065 mg QE/g. The statistical analysis of these results exhibited significant differences among three lettuce varieties (*p* < 0.05), except for the EtOAc extracts of red oak and red coral. The highest TFC content of the red oak and red coral lettuce was found in the EtOH extracts while the highest TFC of butterhead lettuce was found in the hexane extract. Moreover, EtOAc extracts from three lettuce had the lowest TFC content. Previous research also identified TFC in red coral lettuce (21.96 mg QE/100 g fresh weight), butterhead lettuce (3.00 mg QE/100 g fresh weight) (Gan & Azrina 2016) and red oak lettuce (2.67 mg QE/100 g fresh weight) (Mampholo *et al*. 2016). In contrast to our findings, red lettuce had greater TFC than green lettuce. Although the highest flavonoid content was found in the butterhead hexane fraction, we found that ethanol extracts yielded a high amount of flavonoids. This is not surprising since flavonoids can be extracted with both high and low polarity solvents (Mallhi *et al*. 2018).

This study is different from a study by Gan and Azrina (2016), which studied lettuce in Malaysia, in terms of geographical location and types of solvent used. The effect of location may not be able to emphasise since Gan and Azrina used 70% aqueous ethanol for the extraction, but we used 95% ethanol. Another difference is that screening tests were also not reported in that study. We believe that our study yields additional information on qualitative components and antioxidant activities of lettuce.

### Antioxidant Activity

DPPH and ABTS radical scavenging assays are simple and widely used techniques to investigate the antioxidant potential of plant extracts (Sujarwo & Keim 2019). DPPH and ABTS methods have been classified as mixed-mode assays since they require both HAT and ET reactions (Apak *et al*. 2016; Gulcin 2012; Gupta 2015; Li *et al*. 2018; Liu *et al*. 2007; Platzer *et al*. 2021). In this study, DPPH and ABTS assays were used to investigate the IC_50_ from the lettuce extracts (Table 3). Please note that the Pearson correlation between major active compound and their antioxidant activities were not significant since this study was underpowered and did not design to detect such correlation. The IC_50_ values of all fractions in this experiment were higher than those of positive controls, i.e., the antioxidant activity of the three extracts was lower than the positive controls. Table 3 also shows that the IC_50_ values of the EtOH and EtOAc extracts were lower than the hexane extracts. According to the results of total phenolic and flavonoid contents (Table 2), the EtOH and EtOAc extracts exhibited high TPC and TFC which were very proportional to the high antioxidant activities. This agrees with another study indicating that the EtOAc and 95% EtOH extracts contain TPC and TFC which are responsible for the antioxidant activity (Ri *et al*. 2019).

From the DPPH assay, extracts with the strongest to the lowest antioxidants were EtOAc, EtOH and hexane, respectively. We hypothesised that the EtOAc extract had the highest antioxidants because it contained the highest amount of TPC (Table 2). The DPPH assay revealed that the EtOAc fraction of the red coral lettuce had the highest antioxidant capacity. Although the TPC in the EtOAc extract of the red coral lettuce was higher than in the EtOH extract (Table 2), there was no significant difference in the IC_50_ values between the EtOH and the EtOAc extracts (*p* ≥ 0.05). Differences in chemical constituents in the EtOH and EtOAc extracts might play an important role in this phenomenon. Moreover, the EtOAc fractions of red oak and butterhead lettuce had higher antioxidant activity than the hexane and EtOH fractions. DPPH radical scavenging activity of the red coral lettuce in our study (0.325 ± 0.017 mg/mL) was closed to an IC_50_ of 0.304 ± 0.011 mg/mL from 70% ethanolic extract of the red coral lettuce in Malaysia reported by Gan and Azrina (2016). However, the 70% ethanolic extract of the butterhead lettuce reported by Gan and Azrina (2016) had a higher IC_50_ value than the 95% EtOH extract of the butterhead lettuce in this study (4.230 ± 0.402 mg/mL vs. 0.783 ± 0.007 mg/mL, respectively). In addition, from the ABTS assay, the fraction of 95% EtOH contained the highest antioxidant components of three types of lettuce, followed by the fractions of EtOAc and hexane, respectively. The 95% EtOH of the red coral lettuce had the lowest IC_50_ value (0.300 ± 0.002 mg/mL).

Our study did not further investigate carotenoids in the lettuce since carotenoids are lipophilic compounds that can be well dissolved in non-polar solvents (Saini & Keum 2018). Our study used ethyl acetate and 95% ethanol for the extraction which was more polar and not well versed in extracting carotenoids. This is supported by a study showing that when different solvents were used (*n*-hexane, ethyl acetate and ethanol) for the extraction, ethanol extract had the highest antioxidant activity derived from total phenol and flavonoid, without carotenoid contents (Fitriansyah *et al*. 2018). In addition, since carotenoid contents in plants are degraded by drying at 30–50°C (Al-Farsi *et al*. 2005), the heat drying used in our study can degrade carotenoids.

The difference in the number of antioxidants depends on multiple factors. The type of solvent used for extraction is the most important. In general, the secondary metabolites in plants are a mixture of polar and non-polar compounds which can be dissolved in solvents with different polarities. The empirical rule, “like dissolves like”, means that polar molecules dissolve in polar solvents and less-polar molecules dissolve in less-polar solvents (Bhebhe *et al*. 2016). However, the solubility in a solvent is also affected by the type of lettuce, part of use and amount of bioactive compounds (Iloki-Assanga *et al*. 2015). Moreover, the variation of the planting condition such as air, cultivars, harvest, light, minerals, season, soil, temperature and water significantly affected the phenolic and flavonoid contents of plants (Lanza Volpe *et al*. 2021; Liu *et al*. 2007; Zdravković *et al*. 2014). Changes in mineral element content in different areas also affect the nutritional quality and antioxidant activity of lettuce (Sawatdee *et al*. 2021). An example shows that lettuce cultivated in a plant factory with a solution at the lowest mineral concentration yields the highest anthocyanin, polyphenol, and flavonoid. Also, FRAP (ferric reducing antioxidant power) and DPPH activities are the highest in this condition (Song *et al*. 2020).

## CONCLUSION

The three lettuce cultivars (red oak, red coral and butterhead) are good sources of phytochemicals with antioxidant activities including flavonoids, phenolics, coumarins and tannins. Ethanol and ethyl acetate are suitable for extracting antioxidants. We also found that red coral lettuce has the greatest antioxidant activity. Further investigation to increase the antioxidant yields, qualification of the antioxidants, and formulation of the dry extract should be conducted.

## Figures and Tables

**Table 1:** Preliminary phytochemical screening of three lettuce cultivars using the standard procedures.

Test	Results^a^		

	Red oak	Red coral	Butterhead
Flavonoids (Shinoda’s test)	+	+	+
Flavonoids (Molisch’s test)	−	−	−
True tannins	−	−	−
Condensed tannins	−	++	−
Hydrolyzable tannins	++	++	++
Coumarins	++	++	++
Saponins	−	−	−
Anthraquinones	−	−	−
Cardiac glycosides (Kedde’s test)	−	++	++
Cardiac glycosides (Liebermann-Burchard test)	++	++	++
Cardiac glycosides (Keller-Kiliani test)	−	−	−
Phenolics	++	++	++
Alkaloids	−	−	−

*Notes*:

a (++): strong positive result (intense colour); (+): positive result (noticeable colour); (−): negative result

**Table 2:** The percentage yield, total phenolic and flavonoid content of different extracts of red oak (RO), red coral (RC) and butterhead (BH) lettuce.

Extracts^a^	%Yield (%w/w)	Total phenolic content^d^ (mg GAE/g)^b^	Total flavonoid content^d^ (mg QE/g)^c^
ROH	2.08	3.261 ± 0.009	3.145 ± 0.016
ROEA	2.80	5.751 ± 0.008	1.129 ± 0.016^e^
ROE	6.78	4.047 ± 0.002	4.971 ± 0.084
RCH	1.10	1.698 ± 0.004	1.660 ± 0.038
RCEA	2.82	9.747 ± 0.021	1.093 ± 0.075^e^
RCE	5.08	5.646 ± 0.002^d^	3.783 ± 0.106
BHH	1.52	4.169 ± 0.017	7.065 ± 0.005
BHEA	3.22	5.816 ± 0.004	1.441 ± 0.057
BHE	7.42	5.612 ± 0.006^*^	6.659 ± 0.004

*Note*:

a H = hexane, EA = EtOAc, E = 95% EtOH;

b GAE: gallic acid equivalent;

c QE: quercetin equivalent,

d the values in each column are significantly different (*p* < 0.05), the values are expressed as means of triplicate determinations ± SD.

d Total phenolic content of RCE were not significantly different from BHE.

e Total flavonoid content of ROEA were not significantly different from RCEA.

**Table 3:** DPPH and ABTS radical scavenging assays of different extracts of red oak (RO), red coral (RC) and butterhead (BH) lettuce.

Extracts^a^/Standards	DPPH radical scavenging assay^b^ IC_50_ (mg/mL)	ABTS radical scavenging assay^b^ IC_50_ (mg/mL)
ROH	7.457 ± 0.064	11.507 ± 0.013
ROEA	0.549 ± 0.023^c^	0.981 ± 0.009
ROE	3.371 ± 0.026	0.743 ± 0.002
RCH	17.478 ± 0.109	4.555 ± 0.007
RCEA	0.277 ± 0.006^d^	0.398 ± 0.003
RCE	0.325 ± 0.017^d^	0.300 ± 0.002
BHH	5.292 ± 0.057	3.273 ± 0.013
BHEA	0.648 ± 0.011	1.010 ± 0.007
BHE	0.783 ± 0.007^c^	0.783 ± 0.006
Trolox	0.0064 ± 0.0003^e^	0.0049 ± 0.0001
Ascorbic acid	0.0061 ± 0.0003^e^	0.0023 ± 0.0001

*Note*:

a H = hexane, EA = EtOAc, E = 95% EtOH,

b the values in each column are significantly different (*p* < 0.05), the values are expressed as means of triplicate determinations ± SD.

c IC_50_ from DPPH assay of ROEA was not significantly different from BHE.

d IC_50_ from DPPH assay of RCEA was not significantly different from RCE.

e IC_50_ from DPPH assay of Trolox and ascorbic acid was not statistically different.
